# Predicting tumor response and outcome of second-look surgery with ^18^F-FDG PET/CT: insights from the GINECO CHIVA phase II trial of neoadjuvant chemotherapy plus nintedanib in stage IIIc-IV FIGO ovarian cancer

**DOI:** 10.1007/s00259-020-05092-3

**Published:** 2020-11-21

**Authors:** Nicolas Aide, Pauline Fauchille, Elodie Coquan, Gwenael Ferron, Pierre Combe, Jérome Meunier, Jerôme Alexandre, Dominique Berton, Alexandra Leary, Gaétan De Rauglaudre, Nathalie Bonichon, Eric Pujade Lauraine, Florence Joly

**Affiliations:** 1 Nuclear Medicine Department, University Hospital, Caen, France; 2 INSERM 1086 ANTICIPE, Normandie University, Caen, France; 3 Centre Hospitalier Universitaire, Avenue Côte de Nacre, 14000 Caen, France; 4 Medical Oncology, Centre François Baclesse, Caen, France; 5 Surgical Oncology, Institut Claudius Regaud, Toulouse, France; 6 ARCAGY GINECO, Paris, France; 7 Medical Oncology, Hôpital Européen Georges Pompidou, Paris, France; 8 Medical Oncology, CHG, Orléans, France; 9 Medical Oncology, Hôpital Cochin, Paris, France; 10 Medical Oncology, ICO, Nantes, France; 11 Medical Oncology, IGR, Paris, France; 12 Medical Oncology, Institut Sainte Catherine, Avignon, France; 13 Medical Oncology, Clinique Tivoli Ducos, Bordeaux, France

**Keywords:** Ovarian cancer, ^18^F-FDG, PET, PERCIST, MATV

## Abstract

**Background:**

This ancillary study aimed to evaluate ^18^F-FDG PET parameter changes after one cycle of treatment compared to baseline in patients receiving first-line neoadjuvant anti-angiogenic nintedanib combined to paclitaxel-carboplatin chemotherapy or chemotherapy plus placebo and to evaluate the ability of ^18^F-FDG PET parameters to predict progression-free survival (PFS), overall survival (OS), and success of second-look surgery.

**Materials and methods:**

Central review was performed by two readers blinded to the received treatment and to the patients’ outcome, in consensus, by computing percentage change in PET metrics within a volume of interest encompassing the entire tumor burden. EORTC and PERCIST criteria were applied to classify patients as responders (partial metabolic response and complete metabolic response) or non-responders (stable metabolic disease and progressive metabolic disease). Also analyzed was the percentage change in metabolic active tumor volume (MATV) and total lesion glycolysis (TLG).

**Results:**

Twenty-four patients were included in this ancillary study: 10 received chemotherapy + placebo and 14 chemotherapy + nintedanib. PERCIST and EORTC criteria showed similar discriminative power in predicting PSF and OS. Variation in MATV/TLG did not predict PFS or OS, and no optimal threshold could be found for MATV/TLG for predicting survival. Complete cytoreductive surgery (no residual disease versus residual disease < 0.25 cm/0.25–2.5 cm/> 2.5 cm) was more frequent in responders versus non-responders (*P* = 0.002 for PERCIST and *P* = 0.02 for EORTC criteria). No correlation was observed between the variation of PET data and the variation of CA-125 blood level between baseline sample and that performed contemporary to the interim PET, but a statistically significant correlation was observed between ΔSUL_peak_ and ΔCA-125 between baseline sample and that performed after the second cycle.

**Conclusion:**

^18^F-FDG PET using EORTC or PERCIST criteria appeared to be a useful tool in ovarian cancer trials to analyze early tumor response, and predict second-look surgery outcome and survival. An advantage of PERCIST is the correlation of ΔSUL_peak_ and ΔCA-125, PET response preceding tumor markers response by 1 month. Neither MATV nor TLG was useful in predicting survival.

**Trial registration:**

NCT01583322 ARCAGY/ GINECO GROUP GINECO-OV119, 24 April 2012

**Supplementary Information:**

The online version contains supplementary material available at 10.1007/s00259-020-05092-3.

## Introduction

The vast majority of epithelial ovarian cancer (EOC) is diagnosed at an advanced stage and optimal removal of intraabdominal tumor bulk forms a major prognostic factor for survival. In widely spread inoperable cases, the primary treatment may start with neoadjuvant chemotherapy (NACT) followed by interval debulking surgery [1, 2]. In that case, there is some concern to administer bevacizumab during the chemotherapy surrounding the interval debulking surgery, due to the long half-life (14–21 days) of this monoclonal antibody and the interference of anti-angiogenic agents with wound healing. Thanks to a much shorter half-life of 7 to 19 h, nintedanib, an anti-angiogenic tyrosine kinase inhibitor [3–5] might offer a better alternative to bevacizumab in the neo-adjuvant setting.

In addition to the FIGO stage and surgical outcome, the latter being evaluated with various scores such as the Sugarbaker Peritoneal Carcinomatosis index (PCI), the response to platinum-based chemotherapy is a significant prognostic factor [6]. The response to first-line treatment is measured using computed tomography (CT) scan and if the serum tumor marker CA125 is increased at the time of diagnosis, serial CA125 measurements can also be useful in monitoring the treatment response [2].

In clinical trials, an objective evaluation of drug response is essential and new approaches other than CT scan are awaited to better evaluate the objective response to drugs. Treatment response assessment with PET imaging is not included in the current generally accepted guidelines [2]. Therapy assessment with PET can rely on the EORTC PET response criteria [7], released in 1999 or on the PET Response Criteria in Solid Tumors (PERCIST) criteria [8, 9], which were introduced in 2009. Since EOC often presents as a bulky disease and is known to be a heterogeneous disease in terms of expression of various immunohistochemical markers, using volumetric PET metrics such as the metabolic active tumor volume (MATV) or the total lesion glycolysis (TLG) could be of added value [10]. However, unlike PERCIST and EORTC criteria for which thresholds to discriminate between stable, progressive, or responding metabolic disease are known, optimal thresholds for MATV or TLG variation between baseline and post-treatment scans are yet to be determined.

The CHIVA study explored the role of nintedanib in combination with neoadjuvant chemotherapy in unresectable advanced ovarian cancer patients. The aim of this ancillary study of the CHIVA trial was (1) to determine the optimal ^18^F-FDG PET/CT response criteria to be used in EOC to predict overall survival (OS) and progression-free survival (PFS), irrespective of the treatment arm, (2) to explore the capability of ^18^F-FDG PET response to predict outcome of debulking surgery, and **(3)** to correlate PET and serological responses.

## Materials and methods

### Study design and drug administration

This study was a randomized double-blind placebo-controlled phase II trial registered as NCT01583322 ARCAGY/ GINECO GROUP GINECO-OV119. Informed written consent was obtained from each patient.

Eligible were patients with first diagnosis of histological confirmed (cytology alone excluded) epithelial ovarian cancer, fallopian tube or primary peritoneal cancer, FIGO stages IIIc-IV, ECOG performance status < 2, and life expectancy of at least 6 months. Histology had to be obtained by laparoscopy (or by laparotomy), in patients for whom primary debulking surgery had been denied and maximum surgical effort of cytoreduction with the goal of non-residual disease was planned as interval debulking surgery.

Patients were randomized (2:1) to be treated with 3–4 cycles of carboplatin (AUC5) and paclitaxel (175 mg/m^2^) before interval debulking surgery followed by 2 to 3 adjuvant cycles (for a total of 6 cycles) plus either 400 mg daily nintedanib (experimental arm) or placebo at cycles 1 and 2 and 5 and 6 and maintenance therapy for up to 2 years.

The PET ancillary study was optional in the workup of the CHIVA trial, which included a total of 188 patients.

### Site qualification

All patients were scanned on a dedicated, full-ring PET/CT, using the same PET/CT system for the pre- and post-treatment scans. Participating sites were requested to comply with the EANM guidelines for PET tumor imaging [11], especially with regard to cross calibration between the dose calibrator and the PET system, uptake time (60 ± 5 min) and consistency for acquisition and reconstruction parameters for baseline and post-treatment scans. In the case uptake time was not respected for the baseline scan for any logistical problem, centers were requested to keep the same uptake time for the post-treatment examination.

This study involved 9 PET centers and a total of 13 PET/CT systems.

### ^18^F-FDG PET/CT imaging

**Fig. 1:**
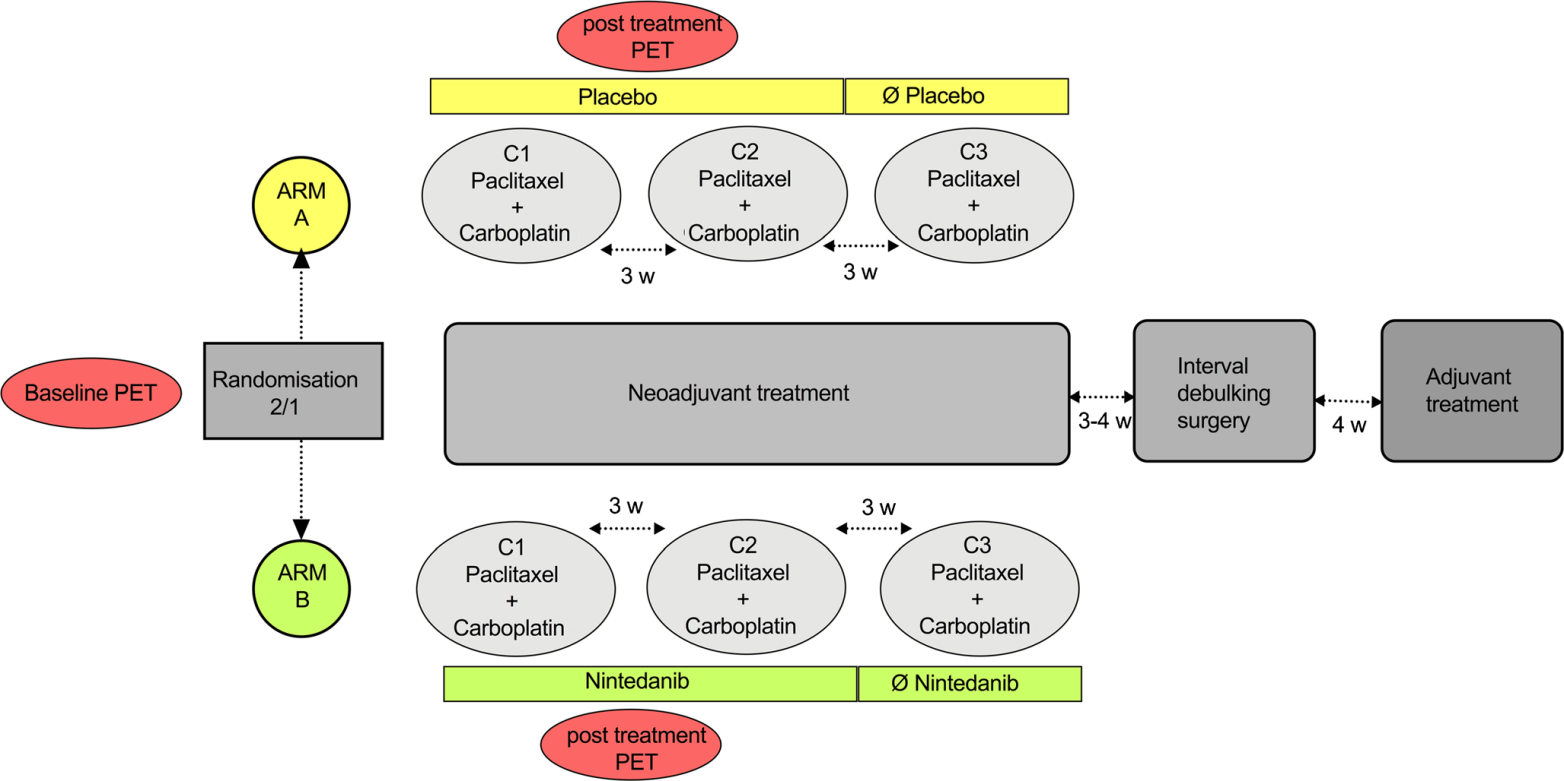
Flowchart description of the study design

^18^F-FDG PET/CT was performed after the first cycle of treatment (Fig. 1).

A uniform imaging protocol was provided to all participating centers, including ^18^F-FDG dosing, uptake time, and plasma glucose recording. Imaging was not performed if plasma glucose was greater than 180 mg/dL.

Patients were scanned from the skull base to the mid-thighs after an intentional 60 ± 5 min of uptake time. Data were corrected for attenuation and scatter events and reconstructed with an iterative algorithm with (*n* = 4) or without point-spread function modeling (*n* = 9). The multidetector spiral CT scans were standard low-dose acquisitions. No intravenous or oral contrast media was used.

### Response monitoring with EORTC and PERCIST criteria

All PET exams were analyzed with central review performed by two PET readers blinded to the received treatment and to the patients’ outcome, in consensus, using MIM software (MIM software Inc., Cleveland, OH). Consistency between the pre- and post-treatment scan for the PET system used, and for acquisition/reconstruction parameters were checked. Patients’ weight, injected activity, and uptake time for the pre- and post-treatment scans were recorded. All these data were extracted from the DICOM headers.

As defined in the PERCIST criteria, the measurable target lesion is the most intense single tumor site on pre- and post-treatment scans, which means that the target lesion is not necessarily the same pre- and post-treatment. In practice, a volume of interest (VOI) was drawn around the tumor lesions, using a SUL threshold above the physiological uptake in the liver. Areas of physiological uptake were manually removed, paying attention to ureteral and bladder physiological excretion. Within this VOI, lean body mass SUL_peak_ (SUL_peak_) and SUL_max_, metabolic active tumor volume (MATV) and total lesion glycolysis (TLG) were automatically measured. Liver background activity (SUL_mean_ liver) was measured in an automatically placed 3 cm diameter sphere in the right lobe.

Based on SUL_peak_ and SUL_max_ variation between the pre- and post-treatment scans, patients were classified according to PERCIST and EORCT PET response criteria as follows:

- *Complete metabolic response* (CMR): complete resolution of ^18^F-FDG uptake in the tumor volume (lower than SUL_mean_ liver and indistinguishable from surrounding blood pool)
- *Partial metabolic response* (PMR): at least 30% (PERCIST) or 25% (EORTC) reduction in tumor uptake
- *Stable metabolic disease* (SMD): less than 30% increase (PERCIST) or 25% (EORTC) or less than 30% decrease (PERCIST) or 25% (EORTC) in tumor 18F-FDG uptake and no new lesions
- *Progressive metabolic disease* (PMD): greater than 30% increase (PERCIST) or 25% (EORTC) in ^18^F-FDG tumor uptake or appearance of new lesions

### Statistical analysis

Data are presented as median and interquartile range.

Survival analyses were performed using univariable Kaplan-Meier survival analyses with log-rank tests to compare survival curves. For PFS and OS, the end-point was defined as the time from diagnosis until relapse or progression, or death as a result of ovarian cancer, respectively.

Concordance between EORTC_25%_ and PERCIST was evaluated using the Cohen’s kappa coefficient.

Prediction of surgical outcome with ^18^F-FDG PET response was assessed by comparing the rate of successful surgery in responders versus non-responders with the Fisher exact test.

Receiver operating characteristic (ROC) curves for PFS and OS were generated to define area under the curve (AUC) and optimal cut-off values of TLG, MATV, and variation in CA-125 blood level between baseline and first or second cycle of treatment, defined as ΔCA125.

Correlation of PET response with variation in tumor blood markers was assessed using the Spearman correlation. For that purpose, variation in PET metrics (SUL_max_, SUL_peak_, MATV, or TLG) after the first cycle of treatment and variation in CA-125 blood level between baseline and first or second cycle of treatment (ΔCA125) were computed as follows:

$$ {\Delta}_{\mathrm{CA}125\ \mathrm{or}\ \mathrm{PET}\ \mathrm{metrics}}=\left(\raisebox{1ex}{$\mathrm{Baseline}-\mathrm{interim}$}\!\left/ \!\raisebox{-1ex}{$\mathrm{baseline}$}\right.\right)\times 100 $$

Though our study was designed to investigate the usefulness of PET response criteria in EOC, we also analyzed baseline and interim PET metrics, used as absolute values, for prediction of survival and outcome of second-look surgery.

Graphs and statistical analysis were performed using GraphPad Prism 8. For all statistical tests, a two-tailed *P* value of less than 0.05 was considered statistically significant.

## Results

### Patients’ demographics

Details regarding FIGO stage, histology, and outcome of surgery after neoadjuvant treatment can be seen in Table 1. More than half of the patients had a complete interval debulking surgery. Post-treatment PET was performed 27 days (21–33.75) following baseline PET examination.

**Table 1:** Patients characteristics

Age	64.5 (58–70.3)	
FIGO stage at randomization	IIIB	0
	IIIC	17
	IV	7
Histological grade	3	18
	2	4
	Unknown	2
Histology	Serous/papillary	22
	Endometroid	1
	Undifferentiated	1
Outcome of surgery	No residual disease	13
	Residual disease < 0.25 cm	3
	0.25 < residual disease < 2.5 cm	1
	Residual disease > 2.5 cm	1
	No surgery	6

### Response monitoring with EORTC PET response criteria

Median (interquartile range) variation of SUL_max_ between baseline and interim PET/CT examinations was − 41.89% (− 23.08/− 58.82).

**Fig. 2:**
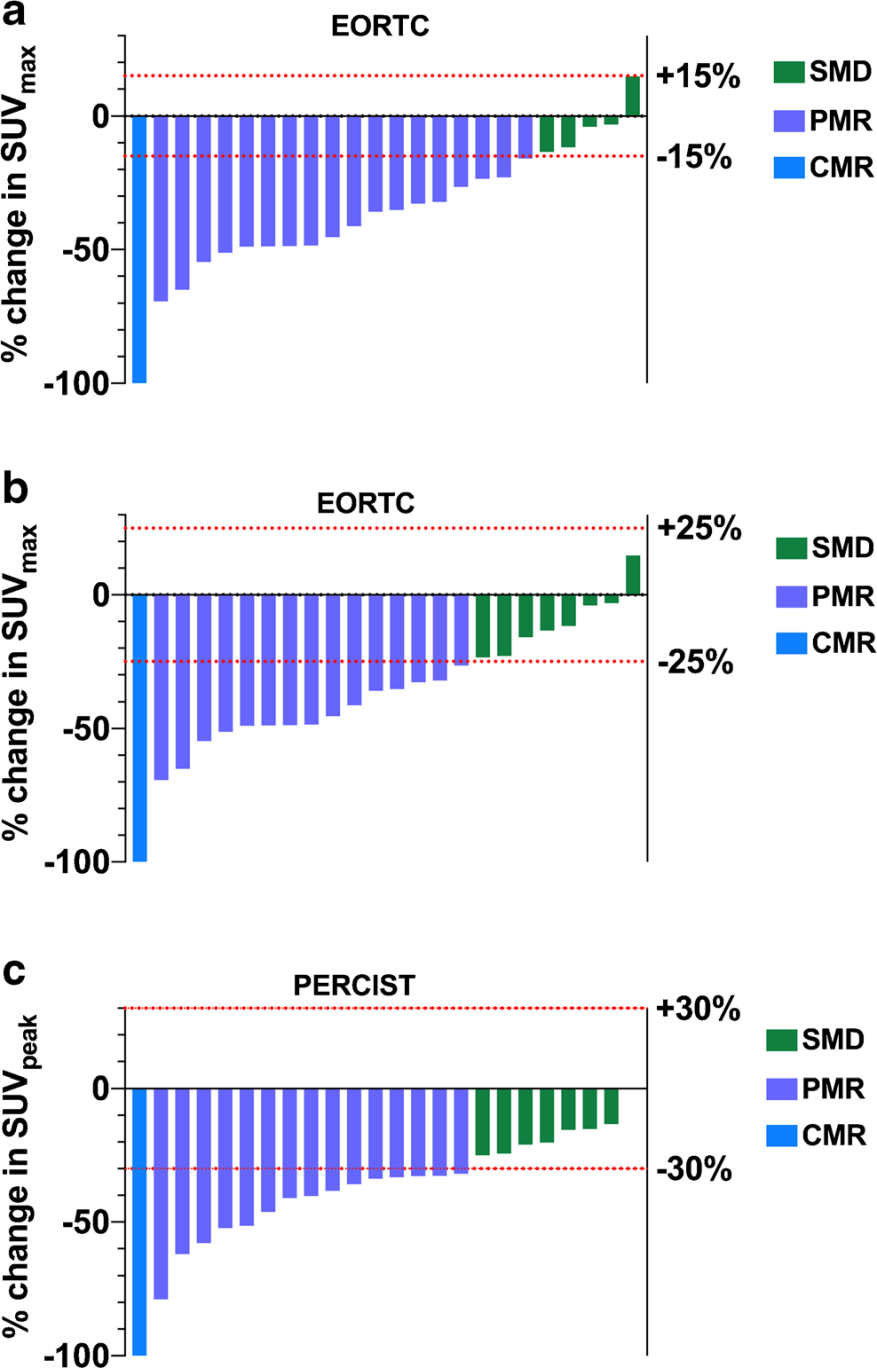
Waterfall diagrams of percentage variation in ^18^F-FDG PET metrics between baseline and interim PET. Panels **a** and **b** display %change in SUL_max_ and panel **c** displays %change in SUL_peak_. Red dotted lines represent threshold used to discriminate between responders and non-responders for EORTC PET response criteria and PERCIST. SMD, stable metabolic disease; PMR, partial metabolic response; CMR, complete metabolic response

**Fig. 3:**
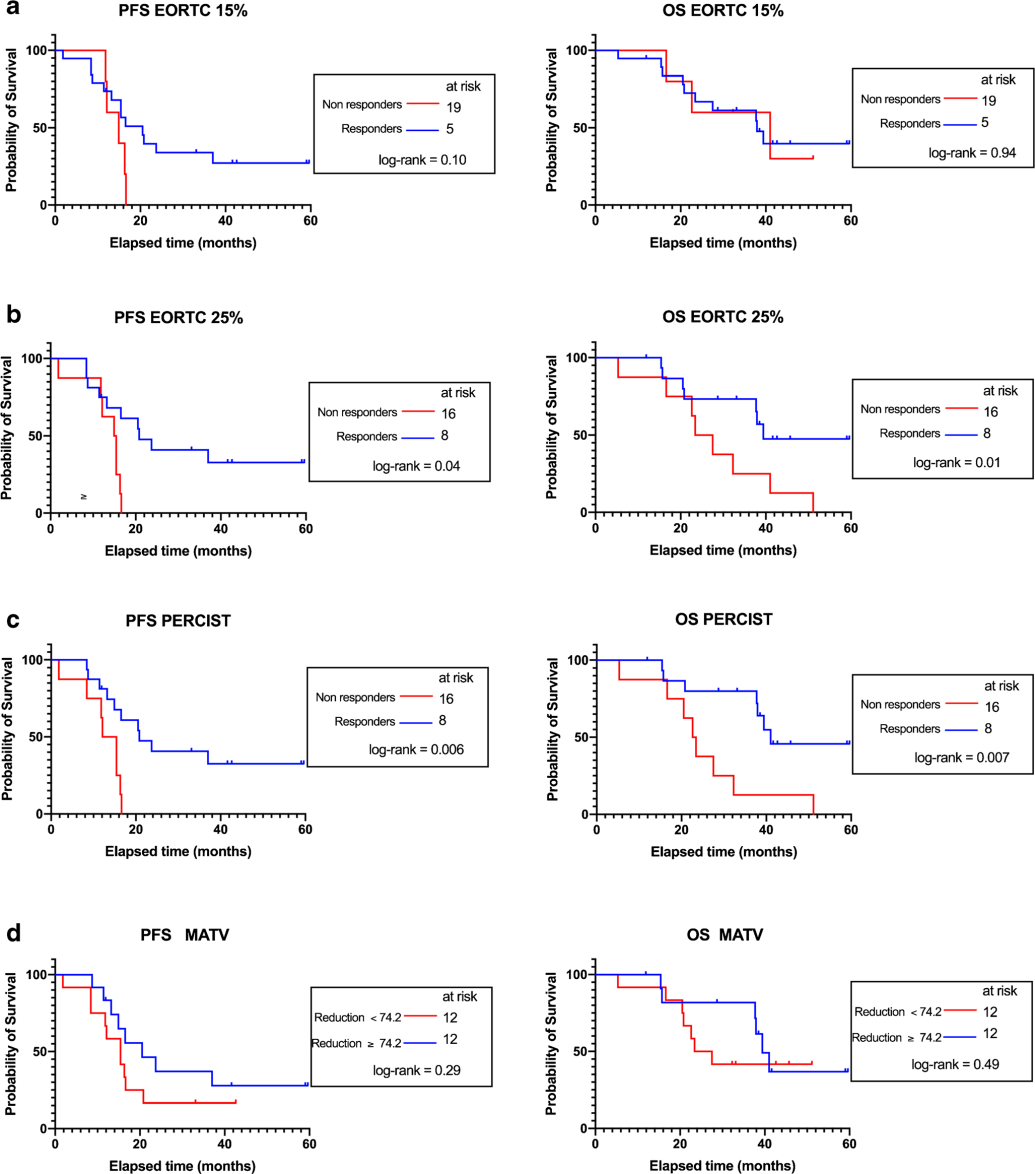
Kaplan-Meier survival curves for progression-free survival (PFS, left panels **a**–**c**) and overall survival (OS, right panels **a**–**c**) in responding (CMR, complete metabolic response or PMR, partial metabolic response) versus non-responding patients (SMD, stable metabolic disease or PMD, progressive metabolic disease) using SUL_max_ (EORTC PET response criteria) and SUL_peak_ (PERCIST). For metabolic active tumor volume (MATV) and total lesion glycolysis (TLG), the thresholds used to discriminate between responders and non-responders were the median values of the series (− 74.2% for MATV and − 78.3% for TLG). MATV and TLG being perfectly correlated, they produced similar results and only the Kaplan-Meier curves for MATV are displayed in panel **d**

Using a 15% threshold for responding lesions, 19 patients were classified as responders (18 PMR and 1 CMR) and 5 patients as non-responders (5 SMD, no PMD was observed) (Fig. 2a). Median progression-free survival (PFS) in responding versus non-responding patients was 20.5 and 14.5 months, respectively (*P* = 0.10). Median overall survival (OS) in responding versus non-responding patients was 37.9 and 41 months, respectively (*P* = 0.94) (Fig. 3a).

Using the 25% threshold, 16 patients were classified as responders (15 PMR and 1 CMR) and 8 patients as non-responders (8 SMD, no PMD was observed) (Fig. 2b). Median PFS in responding versus non-responding patients was 20.8 and 15.15 months, respectively (*P* = 0.01). Median OS in responding versus non-responding patients was 39.4 and 25.45 months, respectively (*P* = 0.04) (Fig. 3b).

### Response monitoring with PERCIST

Overall, compliance to PERCIST requirements regarding variation in injected dose per unit of weight, post injection time, and liver physiological uptake between baseline and post-treatment scans was 88%, 96%, and 75%, respectively (Supplemental Figure 1). Nine out of 24 (37.5%) patients had a target lesion on post-treatment scan different than that of baseline scan.

Median (interquartile range) variation of SUL_peak_ between baseline and interim PET/CT examinations was − 33.49% (− 21.82/− 50.11).

Sixteen patients were classified as responders (15 PMR and 1 CMR) and 8 patients as non-responders (8 SMD, no PMD was observed) (Fig. 2c). Median PFS in responding versus non-responding patients was 20.8 and 13.75 months, respectively (*P* = 0.006). Median OS in responding versus non-responding patients was 41 and 23 months, respectively (*P* = 0.007) (Fig. 3c).

### Concordance between EORTC PET response criteria and PERCIST

Two discordances were observed between EORTC_25%_ and PERCIST: 1 patient classified as SMD by EORTC_25%_ (delta SUL: − 11.7%) and PMR by PERCIST (delta SUL: − 41%), and 1 patient classified as PMR by EORTC_25%_ (delta SUL: − 65%) and SMD by PERCIST (delta SUL: − 20%).

Cohen’s kappa coefficient of concordance between EORTC_25%_ and PERCIST was 0.93.

### Prediction of surgical outcome with PET response

Eighteen out of 24 patients underwent interval debulking surgery. The two patients with discordances in EORTC and PERCIST classifications described above were among the 6 patients who did not undergo surgery. The reasons why surgery was not performed in these patients were severe sepsis, progressive disease, or massive visceral involvement. The CHIVA trial being an intention-to-treat trial [12], these patients were kept in the analysis.

Using either the EORTC_25%_ threshold response criteria or PERCIST, successful surgery was seen more frequently in responders versus non-responders (*P* = 0.02 and *P* = 0.002, respectively).

**Fig. 4:**
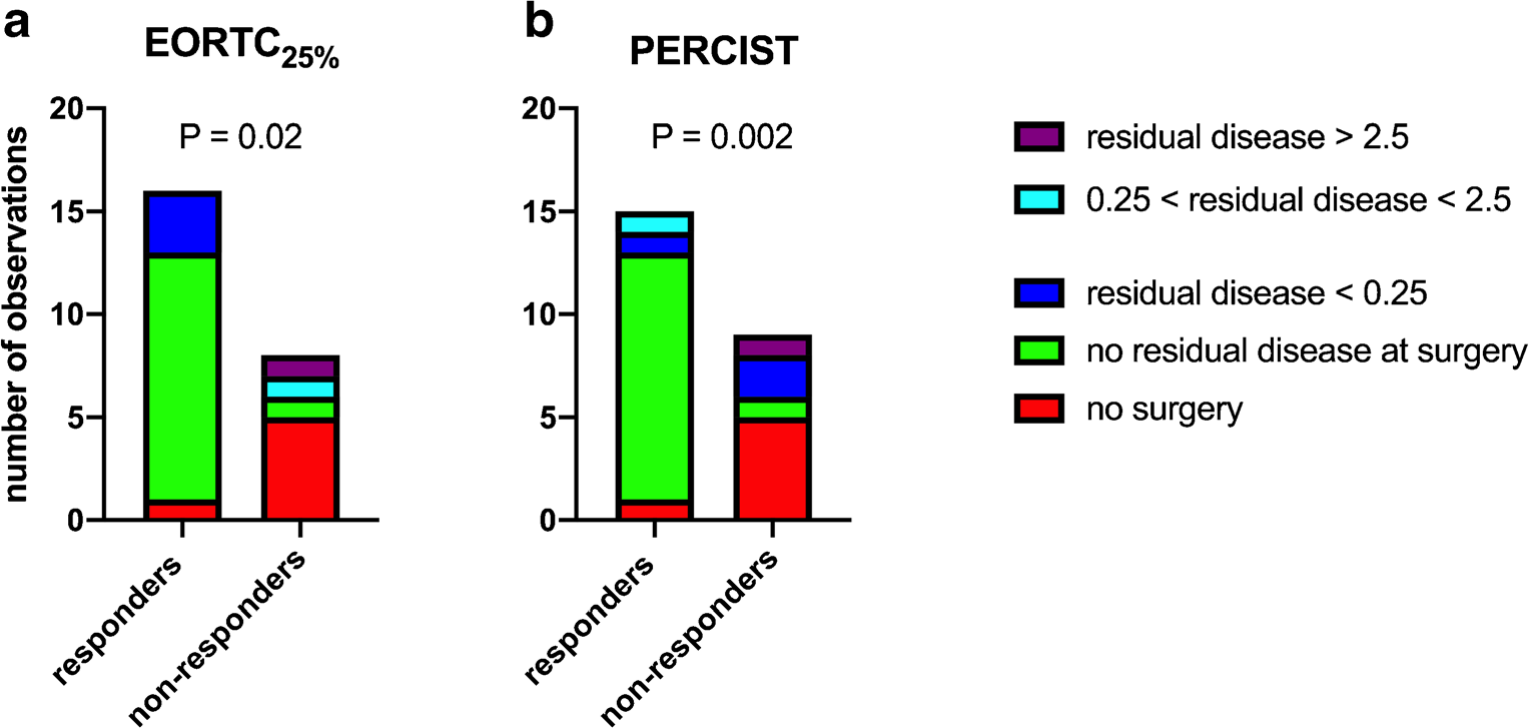
Outcome of surgery in responders and non-responders using EORCT PET response criteria (**a**) or PERCIST (**b**)

Details regarding distribution of the size of tumor residuals in responding versus non-responding tumors for the various type of response evaluation can be seen in Fig. 4.

### Variation of MATV and TLG for therapy monitoring

Median (interquartile range) variation of MATV between baseline and interim PET/CT examinations was − 74.22% (− 44.98/− 90.3). Median (interquartile range) variation of TLG between baseline and interim PET/CT examinations was − 78.3% (− 55.18/− 92.55).

It was proposed in the PERCIST publication that − 40% and + 75% could be used as thresholds to identify responding and progressing tumors when using TLG for therapy assessment. By applying these thresholds proposed therein, 23 patients were classified as responders (22 PMR and 1 CMR) and 2 patients as non-responders (1 SMD, no PMD was observed). These thresholds being poorly discriminative in our series of patients, we used the median values of the percentage variation in MATV and TLG between baseline and interim PET examinations. MATV and TLG being perfectly correlated, they produced similar results (Fig. 3d) with median PFS in responding versus non-responding patients of 20.5 and 15.4 months, respectively (*P* = 0.23). Median OS in responding versus non-responding patients was 39.4 and 25.45 months, respectively (*P* = 0.49).

**Fig. 5:**
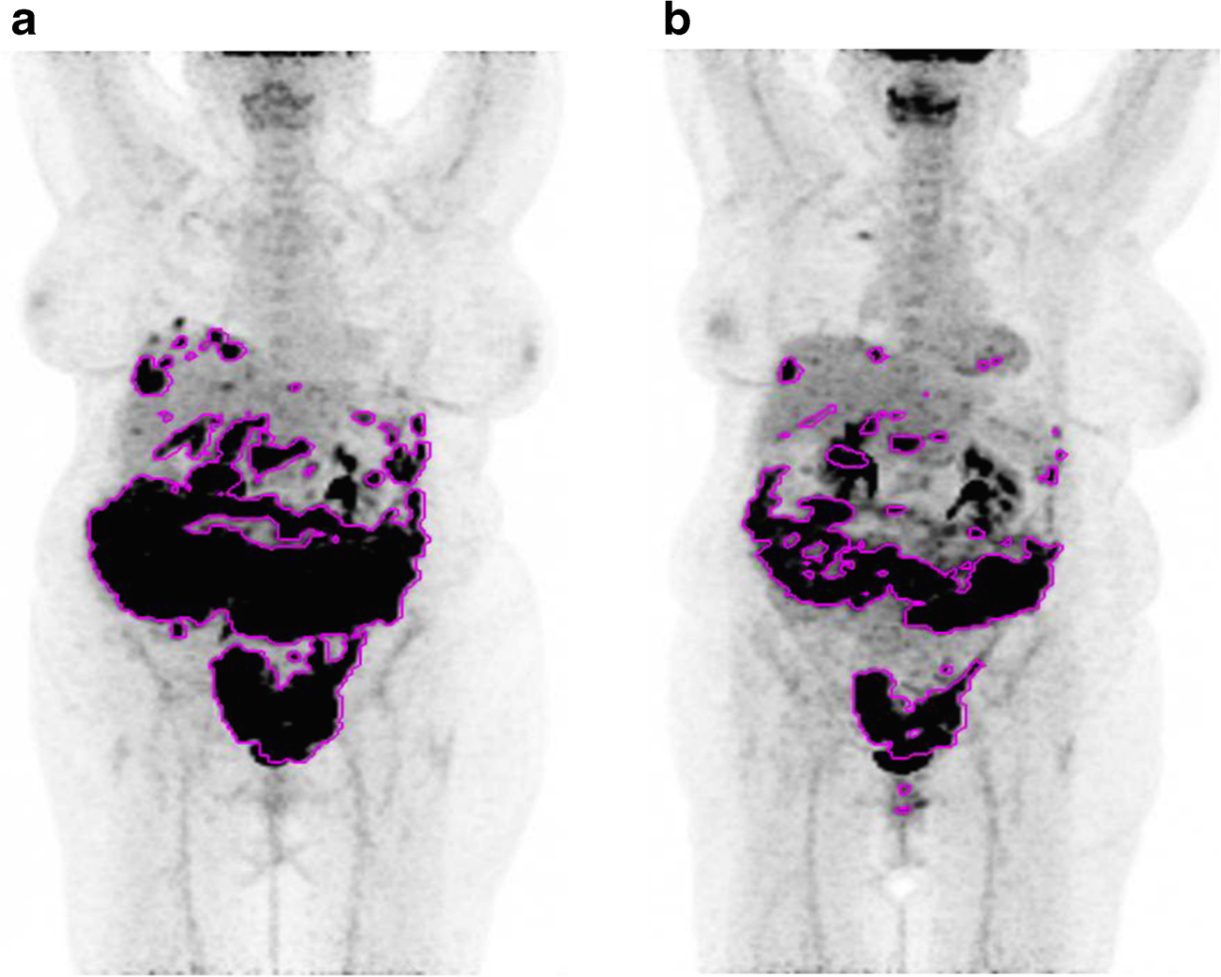
Example of metabolic tumor volume contouring in a patient with bulky peritoneal disease, classified as partial metabolic responder according to EORCT PET response criteria and PERCIST. Note: the uptake visible in the right upper chest is related to a central venous catheter

A representative example of MATV contouring can be seen in Fig. 5.

In addition, since no validated threshold exists regarding the use of either MATV or TLG for response monitoring in solid tumors, a ROC analysis was performed to seek the optimal threshold to predict PFS and OS using these metrics. The areas under the curve were low and statistical significance was not reached (Supplemental Table 1).

### Baseline and post-treatment PET metrics for prediction of survival and outcome of second-look surgery

In addition to the parameters based on interval changes PET parameters, baseline and interim PET metrics (SUV_max_, SUV_peak_, MATV, TLG) were assessed for their predictive values for PFS and OS) by means of a ROC analysis. None of the PET metrics was able to predict survival, though statistical significance was almost reached when seeking the optimal post-treatment SUV_max_ threshold to predict PFS (Supplemental Table 1).

Successful surgery was seen more frequently in patients with low post-treatment MATV and TLG (*P* = 0.02). Baseline PET metrics were not predictive of the outcome of interval surgery.

Details regarding distribution of the size of tumor residuals depending on baseline or interim PET metrics can be seen in Supplemental Figure 2.

### Correlation of PET response with variation in tumor blood markers

**Fig. 6:**
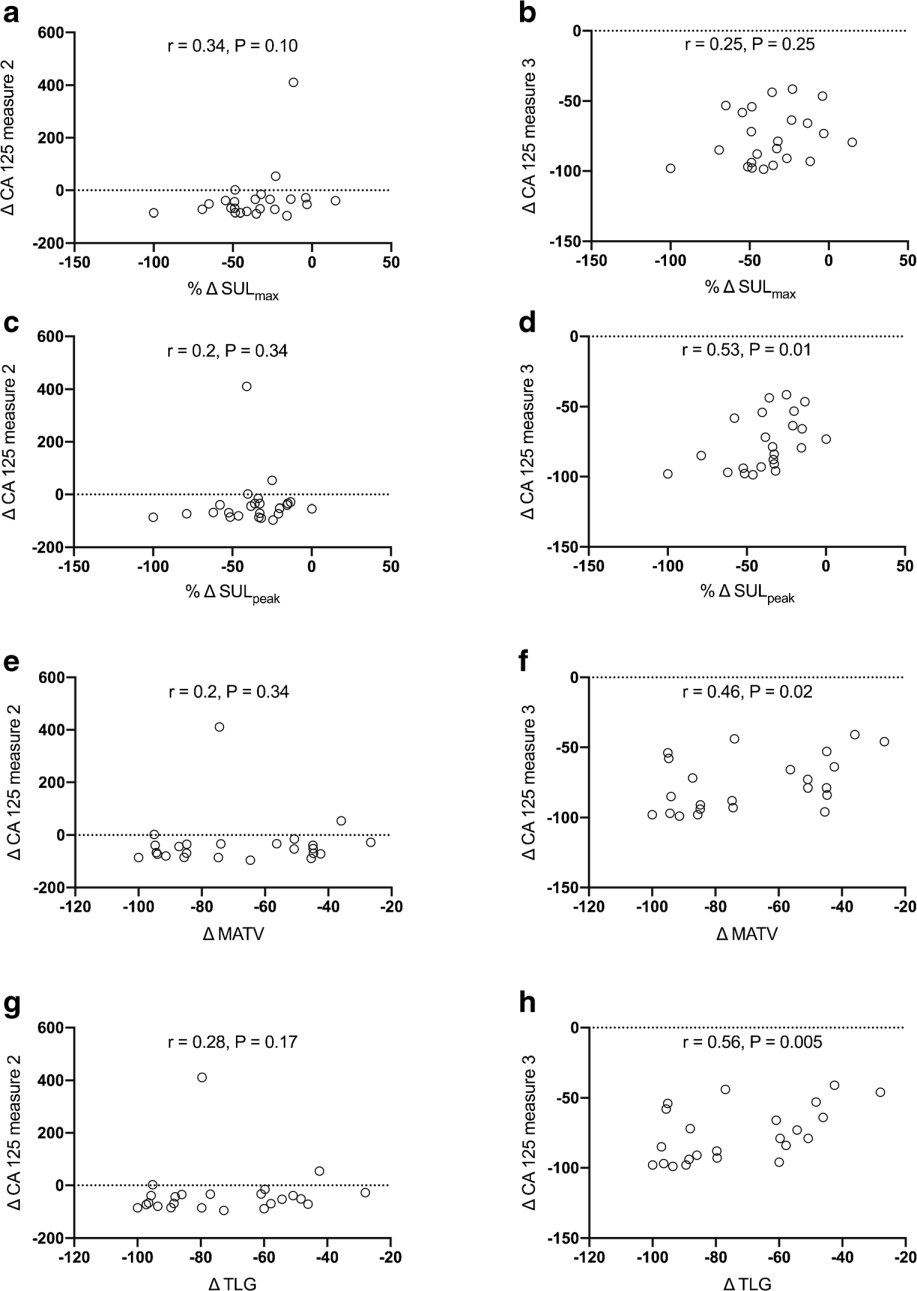
Correlation (Spearman’s coefficient) between the variation of ^18^F-FDG PET metrics (SUL_max_: **a** and **b**, SUL_peak_: **c** and **d**, MATV **e** and **f** or TLG: **g** and **h**) and the variation of CA-125 blood level between baseline sample and that performed contemporary to the interim PET (median of 2 days between PET examination and blood sample, left panels) and that performed after a median of 23 days following the interim PET examination (right panels)

Whatever the PET metrics used (SUL_max_, SUL_peak_, MATV or TLG), no correlation was observed between the variation of PET data and the variation of CA-125 blood level between baseline sample and that performed contemporary to the interim PET (after a median of 2 days) (Fig. 6, left panels). A statistically significant correlation was observed between ΔSUL_peak_, ΔMATV, ΔTLG, and ΔCA-125 between baseline sample and that performed after the second cycle of treatment, CA-125 assays being done after a median of 23 days following the interim PET examination (Fig. 6, right panels).

ROC analysis showed that variation of CA-125 blood levels after the first cycle of treatment was not predictive of survival and that a ΔCA-125 after the 2^d^ cycle greater than − 86% was predictive of PFS (Supplemental Figure 3).

## Discussion

In ovarian cancer clinical trials, an objective evaluation of drug response is essential. Treatment response assessment with ^18^F-FDG PET imaging is not included in the current generally accepted guidelines [2] and literature is scarce for clinical [13] or preclinical data [14].

Our study shows a similar discriminative power in predicting PSF and OS when using PERCIST or EORTC criteria with a 25% threshold value to discriminate between SMD and PMR. Optimal thresholds to discriminate between responders and non-responders with ^18^F-FDG or other PET probes are based on repeatability of tracer uptake within tumors [15, 16]. A recent test-retest study based on double baseline scan in non-small cell lung cancer patients has shown an excellent reproducibility of ^18^F-FDG when performing scan on state-of-art PET systems as per recent guidelines on PET tumor imaging [17], suggesting that variation in SUV greater than 15% would reflect tumor response to treatment. Yet, our study shows that using the 15% threshold recommended by EORTC criteria when assessing tumor response very early in the course of treatment led to the loss of the prognostic value of PET (Fig. 3). In accordance with the literature [18], when using the 25% threshold, a good agreement was found between EORTC PET response criteria and PERCIST.

Since EOC often presents as a bulky disease and is known to be a heterogeneous disease in terms of expression of various immunohistochemical markers and mutational status within different carcinomatosis lesions in the same patients or even within a given nodule, one would assume that using volumetric PET metrics taking into account the whole tumor burden such as MATV or TLG could be useful. That kind of metrics has been shown to be useful in malignant mesothelioma, which as does EOC may present as a bulky disease [10, 19]. In our study, neither the thresholds proposed in the seminal publication on PERCIST [9] to identify responding and progressing tumors when using TLG for therapy assessment nor the use of MATV and TLG median values could predict OS or PFS. In addition, the ROC analysis failed to identify optimal thresholds for MATV and TLG (Supplemental Figure 2). These results differ from those obtained recently by Vallius et al. [20] who reported that a decrease in MATV lesser than 85% allowed to identify patients with stable or progressive disease (as per RECIST 1.1) after neoadjuvant chemotherapy for inoperable EOC with a sensitivity and specificity of 70% and 78%, respectively, and that MTV reduction was associated with PFS. Of note, while this study was focused on PET response criteria, post-treatment MATV and TLG taken as absolute values were predictive of the outcome of interval debulking surgery (Supplemental Figure 2).

Although some more data are required to investigate the potential use of MATV and/or TLG as a tool for therapy monitoring in ovarian cancer, it is noteworthy that in our study, ΔSUL_peak_, which can be easily extracted from PERCIST data, had the same degree of correlation with the variation in CA-125 blood level as that observed with MATV and TLG. Importantly, there is a lag between early PET response and tumor marker response: this correlation was only observed for the tumor marker performed after a median of 23 days after interim PET. Since ΔCA-125 was predictive of survival only after the second cycle of treatment, as opposed to PET response (Supplemental Figure 3), insights from these results are threefold: (i) the lack of correlation between interim PET and contemporary dosage of tumor markers strengthens the potential value of PET as an early surrogate of tumor response, (ii) SUL_peak_, a simple PET metric, performs equally, compared to the time consuming delineation of MATV in a bulky disease often located in the vicinity of high physiological uptake that have to be manually excluded, (iii) this finding is not observed for SUL_max_, giving an advantage to the use of PERCIST over EORTC PET response criteria.

Future research is needed regarding the potential value of combining more complex approaches of tumor marker evaluation, such as mathematical modeling of CA-125 kinetics [21, 22], in combination with PET response.

Our study also shows that early therapy response with ^18^F-FDG PET could be used to predict surgical outcome. Since the two patients with discordances in EORTC and PERCIST classifications described above were among the 6 patients who did not undergo surgery, EORTC and PERCIST showed the same capability in predicting successful surgery, which was seen more frequently in responders versus non-responders. It is noteworthy that the International Collaboration on Cancer Reporting has recommended the use of the chemotherapy response score (CRS) system for the grading of response in EOC [23, 24]. CRS, unlike the success of second-look surgery, is not confounded by many factors. This can be regarded as a limitation of our study. Another limitation of our study is the relatively small number of included patients, which will require confirmation with a larger sample or pooling our data with existing series of patients. However, its strength is the homogeneous advanced stage IIIc-IV FIGO cohort and the statistical significance of the observed results.

## Conclusion

^18^F-FDG PET using EORTC or PERCIST criteria appeared to be a useful tool in ovarian cancer trials to analyze early tumor response, and predict second-look surgery outcome and survival. An advantage of PERCIST over EORTC criteria is the correlation of ΔSUL_peak_ and ΔCA-125, PET response preceding tumor markers response by 1 month. Neither MATV nor TLG were useful in predicting survival.

## Supplementary information

ESM 1 (DOCX 603 kb)
