# Supplementary material for: Predicting tumor response and outcome of second-look surgery with 18F-FDG PET/CT: insights from the GINECO CHIVA phase II trial of neoadjuvant chemotherapy plus nintedanib in stage IIIc-IV FIGO ovarian cancer
Source: Eur J Nucl Med Mol Imaging. 2020 Nov 21;48(6):1998–2008. doi: 10.1007/s00259-020-05092-3 (PMC8113167; doi:10.1007/s00259-020-05092-3)
Supplement: Supplementary file 1 — (DOCX 603 kb) [file 259_2020_5092_MOESM1_ESM.docx]

**Supplemental Table 1**

| Variable | Outcome | AUC | P value |
| --- | --- | --- | --- |
| ΔMATV | PFS | 0.62 | 0.39 |
| ΔMATV | OS | 0.57 | 0.58 |
| ΔTLG | PFS | 0.65 | 0.29 |
| ΔTLG | OS | 0.56 | 0.62 |
| SUV_max_ Baseline | PFS | 0.69 | 0.18 |
| SUV_max_ Baseline | OS | 0.53 | 0.79 |
| SUV_peak_ Baseline | PFS | 0.65 | 0.29 |
| SUV_peak_ Baseline | OS | 0.55 | 0.66 |
| MATV Baseline | PFS | 0.69 | 0.18 |
| MATV Baseline | OS | 0.56 | 0.62 |
| TLG Baseline | PFS | 0.62 | 0.29 |
| TLG Baseline | OS | 0.52 | 0.84 |
| SUV_max_ Interim | PFS | 0.73 | 0.09 |
| SUV_max_ Interim | OS | 0.50 | 0.98 |
| SUV_peak_ Interim | PFS | 0.69 | 0.18 |
| SUV_peak_ Interim | OS | 0.51 | 0.93 |
| MATV Interim | PFS | 0.67 | 0.23 |
| MATV Interim | OS | 0.57 | 0.58 |
| TLG Interim | PFS | 0.69 | 0.16 |
| TLG Interim | OS | 0.56 | 0.62 |

Supplemental Table 1: results of ROC analysis aiming at predicting survival (progression-free survival, PFS; overall survival, OS) using baseline and post-treatment PET metrics.

**Supplemental Figure 1**

**
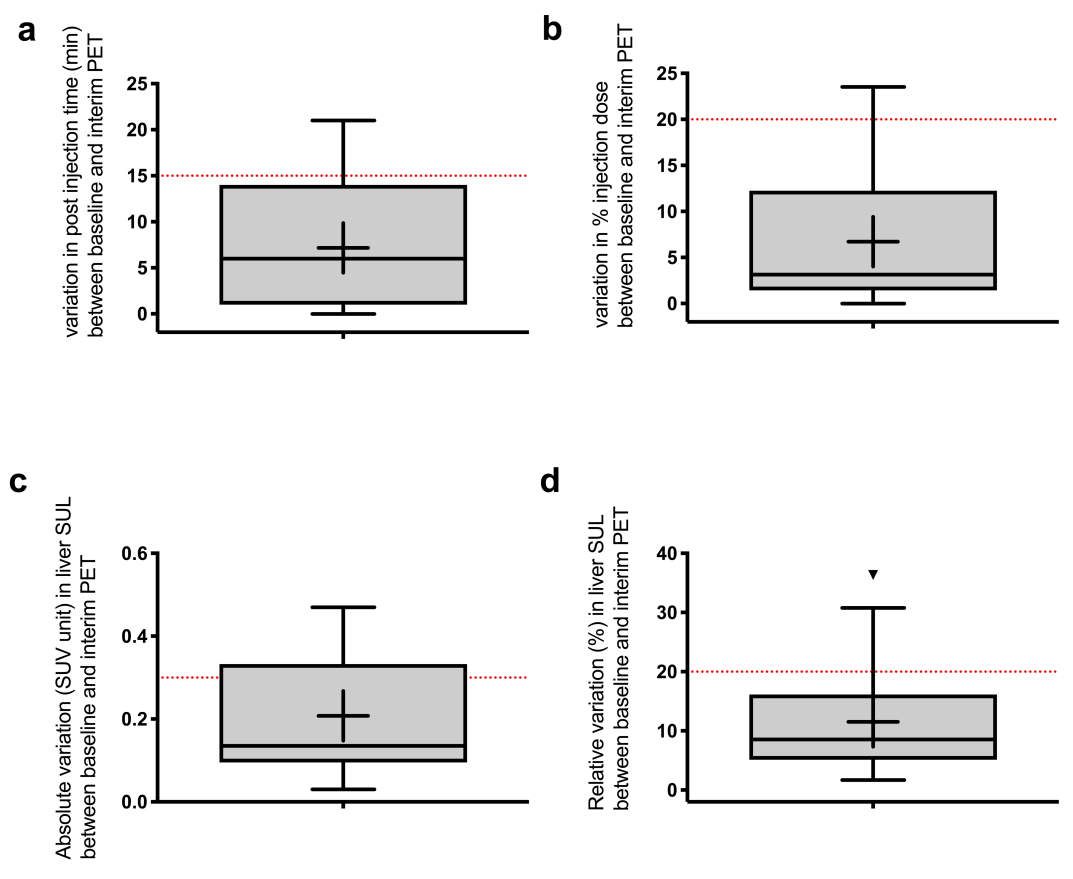
**

**Supplemental Figure 1:** compliance to PERCIST requirements regarding variation in uptake time (**a**), injected dose (**b**) and liver uptake (absolute variation: **c**; relative variation: **d**) between baseline and interim PET. Data are shown as Tukey box: lines represent median and interquartile range, cross represents mean). Red dotted lines show limit of acceptance for PERCIST.

**Supplemental Figure 2**

**
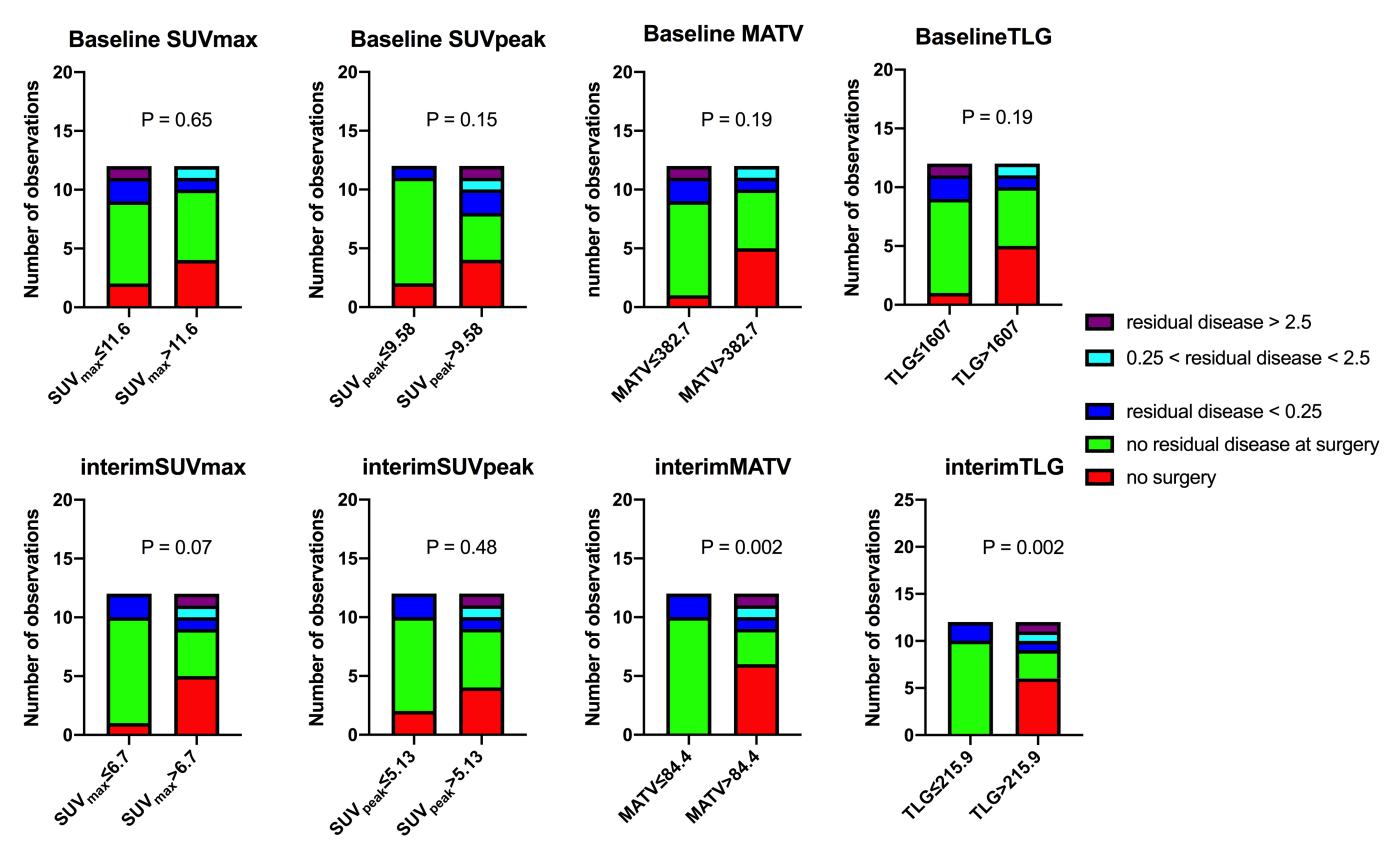
**

**Supplemental Figure 2:** Outcome of surgery depending on various baseline and post-treatment PET metrics.

**Supplemental Figure 3**

**
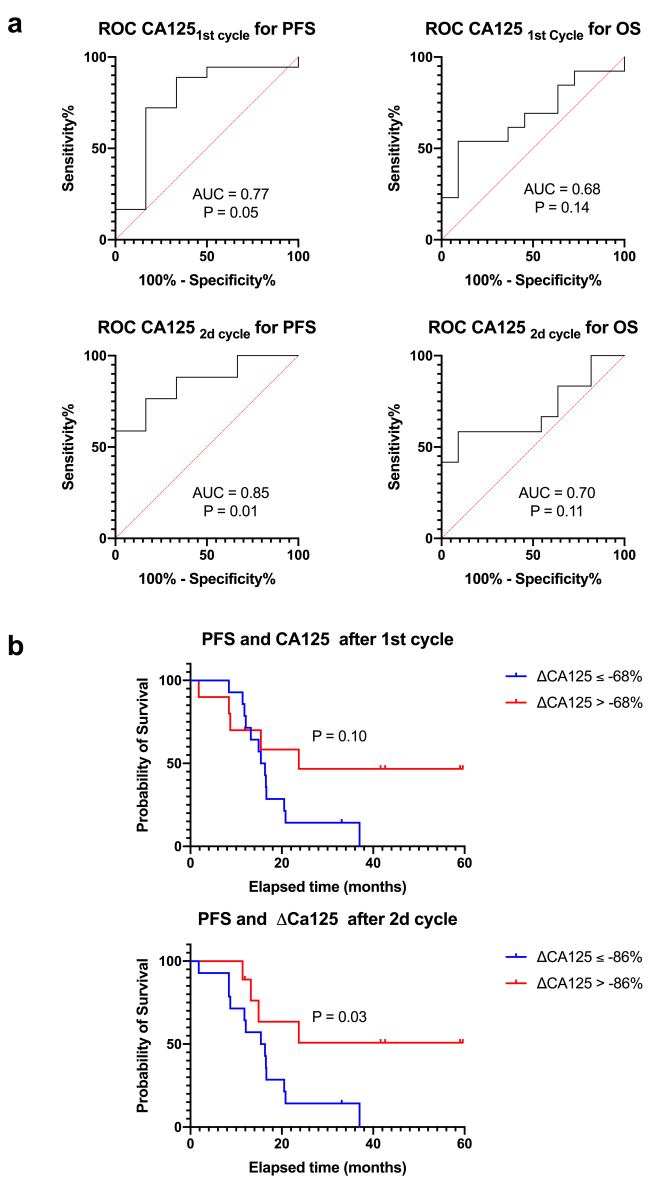
**

**Supplemental Figure 3:** Receiver operating characteristic (ROC) curves for PFS and OS were generated in an attempt to define area under the curve (AUC) and optimal cut-off values of variation in CA125 blood levels between baseline and post-treatment samples, in order to predict survival (**a**). Kaplan-Meier survival curves for progression-free survival (PFS) and overall survival (OS) in patients in whom a decline in CA125 greater than thresholds determined in panel **a** (**b**).
